# Long-Lasting Enhancement of Visual Perception with Repetitive Noninvasive Transcranial Direct Current Stimulation

**DOI:** 10.3389/fncel.2017.00238

**Published:** 2017-08-15

**Authors:** Janina R. Behrens, Antje Kraft, Kerstin Irlbacher, Holger Gerhardt, Manuel C. Olma, Stephan A. Brandt

**Affiliations:** ^1^Charité Universitätsmedizin Berlin Berlin, Germany; ^2^NeuroCare Clinical Research Center, Charité Universitätsmedizin Berlin Berlin, Germany; ^3^Department of Psychiatry, Psychiatric University Hospital St. Hedwig, Charité Universitätsmedizin Berlin Berlin, Germany; ^4^Medical care center, MVZ Reinickendorf Berlin Berlin, Germany; ^5^Center for Economics and Neuroscience, Rheinische Friedrich-Wilhelms-Universität Bonn Bonn, Germany

**Keywords:** contrast sensitivity, noninvasive brain stimulation, plasticity, transcranial direct current stimulation, visual perceptual learning, primary visual cortex

## Abstract

Understanding processes performed by an intact visual cortex as the basis for developing methods that enhance or restore visual perception is of great interest to both researchers and medical practitioners. Here, we explore whether contrast sensitivity, a main function of the primary visual cortex (V1), can be improved in healthy subjects by repetitive, noninvasive anodal transcranial direct current stimulation (tDCS). Contrast perception was measured via threshold perimetry directly before and after intervention (tDCS or sham stimulation) on each day over 5 consecutive days (24 subjects, double-blind study). tDCS improved contrast sensitivity from the second day onwards, with significant effects lasting 24 h. After the last stimulation on day 5, the anodal group showed a significantly greater improvement in contrast perception than the sham group (23 vs. 5%). We found significant long-term effects in only the central 2–4° of the visual field 4 weeks after the last stimulation. We suspect a combination of two factors contributes to these lasting effects. First, the V1 area that represents the central retina was located closer to the polarization electrode, resulting in higher current density. Second, the central visual field is represented by a larger cortical area relative to the peripheral visual field (cortical magnification). This is the first study showing that tDCS over V1 enhances contrast perception in healthy subjects for several weeks. This study contributes to the investigation of the causal relationship between the external modulation of neuronal membrane potential and behavior (in our case, visual perception). Because the vast majority of human studies only show temporary effects after single tDCS sessions targeting the visual system, our study underpins the potential for lasting effects of repetitive tDCS-induced modulation of neuronal excitability.

## Introduction

Sensitivity to contrast is crucial, not only for vision at dusk and nighttime (Brabyn et al., 2005), but also in daylight, e.g., while reading. The processing of visual contrast is one of the main functions of the primary visual cortex (V1; Foster et al., 1985; Mullen et al., 2010), which plays a key role in humans' faculty to visually process their environment. V1 receives input from the retina via the optic nerve, processes visual perceptual information, and is the basis for further integration of visual information in higher visual and nonvisual areas (Fahle, 2004; Schummers et al., 2005). While the functions of V1 are relatively well understood, the degree of plasticity of the human visual cortex is still largely unclear. Exploring the neuroplastic capacity of V1 in the intact human brain may help us understand its ability to recover from damage.

Given the increasing average life expectancy of large parts of the world's population and the fact that stroke occurs more frequently in the elderly (Feigin et al., 2014), often affecting the visual cortex, potential treatments with the aim of rehabilitating the visual system are needed.

Previous studies have shown that the neuronal configuration of visual cortices can be altered by repeatedly performing visual tasks. Training on such tasks has been demonstrated to lead to a short-term or permanent improvement of vision: Several animal studies provide evidence that visual perceptual learning (PL) is associated with a change of local neuronal inhibition and excitation networks within V1 layers, which occurs under modulation from higher brain cortices (top-down) and vice versa (bottom-up; for an overview see Foster et al., 1985; Schummers et al., 2005).

In humans, Sowden et al. (2002) observed that improved contrast perception in a sinusoidal luminance gratings task prevailed for 6 months after extensive practice. Similarly, functional magnetic resonance imaging (fMRI) studies have shown that several weeks of training in a texture discrimination task (Yotsumoto et al., 2008) or in low-contrast oriented patterns (Furmanski et al., 2004) led to improved task performance that was paralleled by an enhanced blood oxygen level-dependent (BOLD) signal in V1, indicating neuronal plasticity in progress (Maertens and Pollmann, 2005).

Together, these studies provide evidence for the neuroplasticity of human V1. However, from a therapeutic perspective, the applicability of extensive training to induce learning processes as mentioned above is limited because they are very time-consuming and demanding for the patient. Therefore, exploration of the lasting effects of brain stimulation has the potential to provide a basis for the development of a practical and time-saving therapeutic tool that enhances visual performance in patients.

Transcranial direct current stimulation (tDCS) is a noninvasive brain stimulation technique (NIBS) that has comparable effects to those of rhythmic stimulation techniques (Lang et al., 2007). For instance, in a study by Clavagnier et al. (2013), continuous theta burst stimulation of the visual cortex temporarily improved contrast sensitivity in adults with amblyopia. A previous tDCS study by Antal et al. (2004) showed a change in the time to the N70 peak of the primary visual evoked potential (VEP), indicating tDCS-induced changes related to oscillatory activity.

Plow et al. (2011, 2012) applied anodal tDCS to patients with stroke concurrent to vision restoration therapy (VRT) and observed superior expansion of the visual field compared to VRT alone. Olma et al. (2013) found that patients with stroke with occipital lesions showed improved motion perception (a function attributed to V5) after anodal tDCS over the unimpaired V1, which was still measurable up to 28 days later.

A channel through which tDCS possibly works is that tDCS could improve visual perception by enhancing the previously described inert neuronal plasticity of V1. Regarding the level of individual neurons, it is possible that immediate tDCS effects are generated by the modulation of cortical activity in neocortex cells through shifting the resting membrane potential (for an overview see Nitsche et al., 2008). Longer-term effects of repeated tDCS might result from synaptic strengthening that is triggered by the increased short-term activity and that is similar to the consolidation of learned visual performance (for an overview see Sale et al., 2011). Thus, on a cellular level, both the consolidation of learned visual performance (Sale et al., 2011) and tDCS (Nitsche et al., 2008) involve long-term potentiation-dependent synaptic strengthening.

Until now, studies that investigated the effects of single tDCS application in the visual system showed only weak tDCS effects compared to other brain areas (for an overview, see Antal et al., 2011).

We here investigate the hypothesis that repetitive application of anodal tDCS to the visual cortex changes contrast perception in healthy subjects. This poses a challenge in that it requires improving an already physiologically intact working visual system. In contrast to previous studies, we investigate long-term effects that result from repeated tDCS application alone, without any concurrent training in a psychophysical, behavioral task. More specifically, this study investigates whether it is possible to boost contrast perception in healthy subjects for an extended period of time. This, in turn, could serve as a basis for further clinical research regarding the plasticity of this area.

## Methods

### General setup

**Figure 1:**
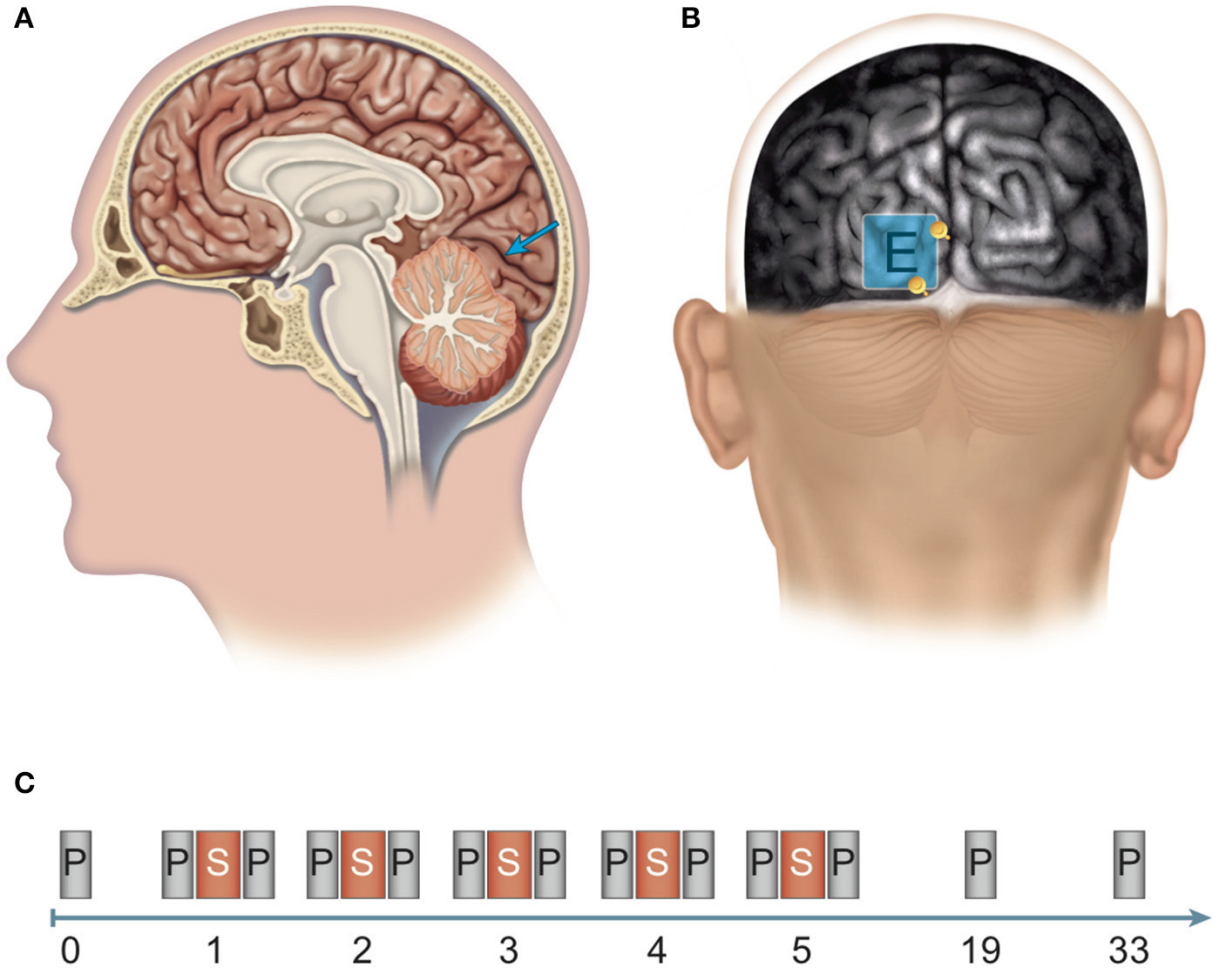
Site of stimulation and experimental design. **(A, B)** Determination of the position of the polarization electrode: An anatomical magnetic resonance image (MRI) was aligned to the surface using the Nexstim system for MRI-guided brain stimulation. **(A)** The target region for anodal stimulation was the left-hemispherical striate area (V1, blue arrow). **(B)** The polarization electrode (E) was placed over the target region and between two anatomical structures (see yellow depth markers): laterally, the inter-hemispherical fissure, and caudally, the boundary between the occipital cortex and the cerebellum (cerebellar tentorium). **(C)** Experimental design: Pre-test before the experiment (0). Stimulation phase: Over five consecutive days (days 1–5), subjects received brain stimulation (S) via anodal transcranial direct current stimulation (tDCS) or sham stimulation and participated in computerized threshold perimetry tests before and after stimulation. Follow-up measurements: Computerized threshold perimetry measurements were taken 2 and 4 weeks (days 19 and 33, respectively) after the final stimulation. *N* = 24 healthy subjects (12 sham group, 12 anodal group).

We applied anodal tDCS or sham stimulation for 20 min on 5 consecutive days to V1 of 12 randomly chosen subjects (anodal group) and 12 control subjects (control group), respectively (Figure 1). High contrast sensitivity is defined as the ability to detect even minor differences in the luminance of visual input. Contrast sensitivity in different parts of the human visual field can be measured by automated, computer-based threshold perimetry, which measures contrast sensitivity in decibels (dB). Small dots of light of varying luminance were presented repeatedly on an isoluminous background at different locations within the central 10° of the visual field. When applying this method, improved contrast sensitivity is characterized by a decrease in the stimulus luminance that is necessary for the presented stimuli to be just above the detection threshold, given the background luminance.

### Subjects and design

Twenty-four healthy, right-handed subjects with a mean age of 24.5 years (*SD* = 3.53) participated. All subjects provided written informed consent prior to their participation in the study. The 24 subjects were randomly assigned to the anodal tDCS group or to the control group. Because of a possible gender-specific difference in modulatory effects of anodal tDCS on V1 (Chaieb et al., 2008), our experiment comprised 12 women and 12 men and a homogeneous distribution of 6 women and 6 men in each group. Included were subjects with normal or corrected-to-normal visual acuity and no known neurological, psychiatric, or ophthalmic impairments. We used a double-blind, sham-controlled, between-subjects design. The study was approved by the ethics committee of the Charité – Universitätsmedizin Berlin in conformity with the tenets of the Declaration of Helsinki. At the end of our experiment, all subjects were paid for their participation.

### Stimulation technique and procedure

tDCS was delivered by a battery-driven DC-stimulator (DC-STIMULATOR PLUS, NeuroConn GmbH, Ilmenau, Germany). We chose the electrode position based on electrode positions of previous tDCS studies on the visual system (Antal et al., 2011) that had proven suitable in our laboratory (Kraft et al., 2010). The reference electrode (size: 7 × 5 cm) was placed on the middle of the skullcap, i.e., at position Cz. It has been shown that binocular viewing may impair the effects of NIBS on a visual perceptual task (Saint-Amour et al., 2005). This might be explained by providing a more robust cortical representation of the visual stimuli (Meese et al., 2006). Therefore, our participants viewed the stimuli monocularly with a patch over their nondominant (left) eye.

The occiput side was randomly chosen (left occiput). To facilitate group analysis, we consistently applied tDCS to the chosen (left) visual cortex of all subjects. To ensure accurate placement of the polarization electrode, positioning was guided by using T_1_-weighted magnetic resonance (MR) imaging (MP-RAGE sequence, TR/TE = 10/4 ms, FA = 12°, TI = 100 ms, voxel size = 1 × 1 × 1 mm^3^). The MR image was acquired with a 1.5-Tesla MAGNETOM Vision MR scanner (Siemens, Erlangen, Germany). The MRI data were aligned to the scalp via the navigated brain stimulation system eXimia NBS System 3.0 (Nexstim Germany GmbH, Frankfurt, Germany). We placed the middle of the electrode over the striate area of the primary visual cortex of the left hemisphere (see Figure 1).

tDCS has been shown to be a safe method, and it is suitable for double-blind experimental protocols (Gandiga et al., 2006). Conforming to current safety guidelines and recommendations (Poreisz et al., 2007), both electrodes were applied in saline-soaked synthetic sponges in order to reduce impedance. Electrodes were fixed on subjects' heads with self-adhesive bandages. Subjects received anodal tDCS (1.5 mA, current density: 0.06 mA/cm^2^) or placebo (sham) stimulation of the left visual cortex for 20 min on 5 consecutive days. The stimulation protocol featured automatic ramping at both the beginning and the end of the stimulation for 15 s. The sham stimulation procedure was identical, but current application was automatically limited to ramping times. This ensured that subjects could not distinguish real from sham stimulation. This method was utilized in previous studies from our laboratory (Kraft et al., 2010).

### Measurement of contrast sensitivity

Before and immediately after stimulation, contrast sensitivity of subjects was measured using computerized threshold perimetry (Humphrey Field Analyzer II, Carl Zeiss Meditec, Inc., Dublin, CA). The measurements were performed using a 10-2 strategy, i.e., including the central 10° of the visual field in steps of 2° [Swedish Interactive Threshold Algorithm (SITA)] (Bengtsson et al., 1997). During measurement, subjects were seated in a dark room at a distance of 30 cm to the presented light stimuli. Stimuli were shown with varying luminance for 200 ms (stimulus size: Goldmann III; constant background illumination: 10 cd/m^2^). Subjects had to push a button every time they detected a light stimulus. They received no feedback and had been accustomed to the measurement procedure before participating in the experiment. All measurements were made under fixation control. At the end of each measurement, the stimulus threshold was calculated for 68 visual field positions within the central 10° of the visual field of the right eye (the left eye was covered).

To evaluate potential long-lasting effects, subjects completed the 10-2 threshold perimetry measurement at two follow-up dates (no tDCS application), 2 (day 19) and 4 weeks (day 33) after the last day of stimulation (day 5; see Figure 1).

### Analysis and statistics

Statistical analysis was performed using SPSS, version 19 (IBM Corp., Armonk, NY). An analysis of variance (ANOVA) with repeated measures was performed to investigate tDCS effects during the stimulation phase (days 1–5) and follow-up dates (days 19 and 33). Our hypothesis was that anodal tDCS would enhance contrast sensitivity (measured as higher dB) compared to sham stimulation over the stimulation phase. Therefore, the independent variable was tDCS stimulation (between-subject factor *stimulation*). The within-subject factors were (a) the effect between before and after stimulation, defined as *intervention*, (b) the development over time (factor *time*) and (c) the degrees of the visual field, defined as *eccentricity*. Subsequent exploratory tests (ANOVAs and unpaired *t*-tests) were performed exploratively (no α correction) to analyze the pattern of the development of contrast sensitivity of all subjects (anodal and sham stimulation). The Greenhouse–Geisser correction was applied when appropriate. All tests were two-tailed, and significance for all effects was assumed when *P* < 0.05. Contrast sensitivity was the dependent variable in all analyses.

## Results

### Repeated anodal tDCS improves contrast sensitivity

**Figure 2:**
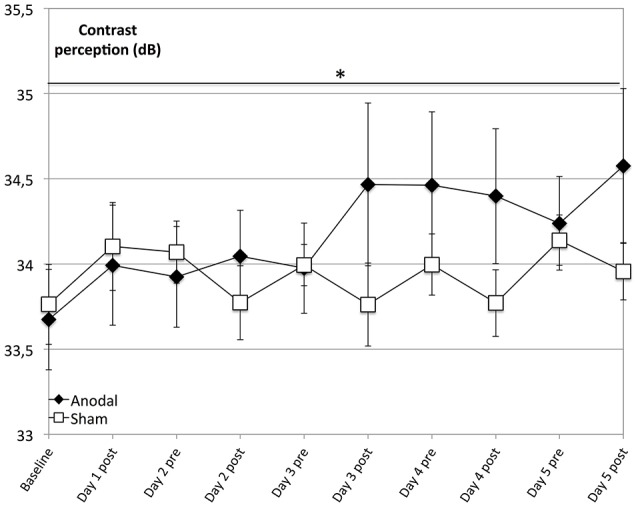
Effects of anodal and sham tDCS on 5 consecutive days. Contrast perception improved across both groups and was not significant in the control group (rectangle line; within-subject effect *time*: *F* = 2.782, *P* = 0.066). Effects were greater for the anodal group (diamond line; interaction of within-subject effect *intervention* and between-subject effect *stimulation*: *F* = 6.456, *P* = 0.019), with a significantly greater average increase in performance over time (days 1–5, interaction of within-subject effect *time* and between-subject effect *stimulation*: *F* = 2.237, *P* = 0.037). ^*^P < 0.05, error bars show standard error of the mean (SEM). *N* = 24 healthy subjects (12 sham group, 12 anodal group).

The main finding of our analysis is that compared to sham, the anodal group showed a significantly greater enhancement of contrast perception (Figure 2; days 1–5, *P* = 0.037). Although we observed enhancement of contrast perception across both groups, it was not significant in the sham group (*P* = 0.066).

**Figure 3:**
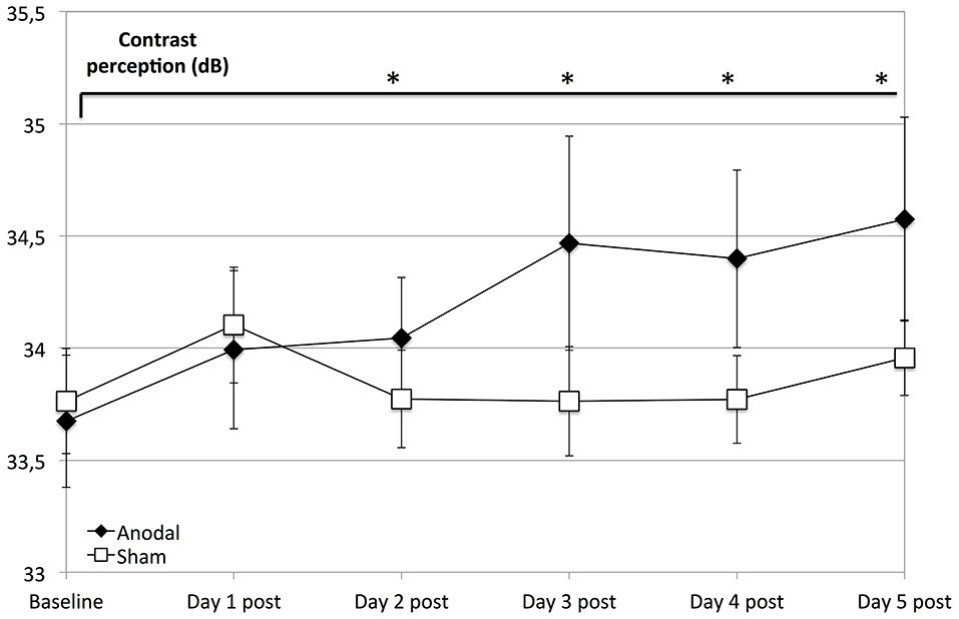
Effects of anodal and sham tDCS at 5 consecutive days after stimulation. Compared to the control group (rectangle line), effects of stimulation were greater for the anodal group at days 2–5 compared to baseline (ANOVA days 1–33, days 19 and 33 are not shown) diamond line; contrasts of within-subject effect *time* and between-subject effect *stimulation*: day 2: *F* = 4.659, *P* = 0.042; day 3: *F* = 9.293, *P* = 0.006; day 4: *F* = 6.972, *P* = 0.015; day 5: *F* = 6.332, *P* = 0.020). ^*^P < 0.05, error bars show standard error of the mean (SEM). *N* = 24 healthy subjects (12 sham group, 12 anodal group).

Regarding the development at single days as a comparison from baseline (as day 1 pre stimulation) to days 1–5 post-stimulation (Figure 3), the anodal group showed a significantly greater enhancement of contrast perception (compared to sham) from day 2 to 5 (*P* < 0.050).

### Contrast perception remains high 24 h after tDCS stimulation

This analysis revealed that contrast perception increased more between days for the anodal group than for the sham group. Average 24-h effects (d*n*pre − d(*n*−1)pre) were significantly greater in the anodal group, compared to sham (between-subject stimulation: *F* = 6.332, *P* = 0.020).

### tDCS effects are based on significantly greater immediate enhancement

**Figure 4:**
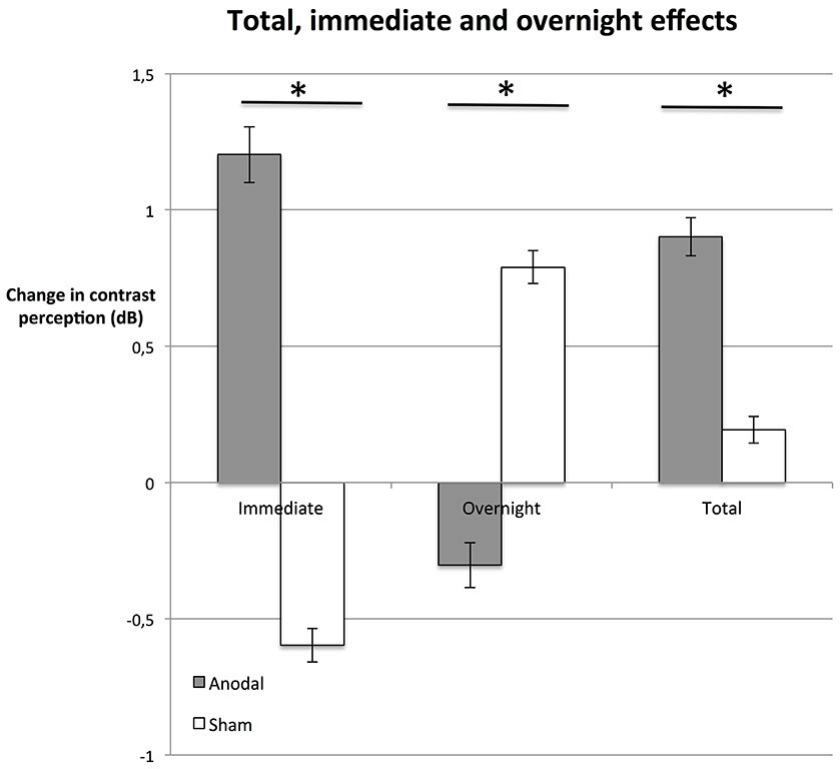
Immediate, overnight, and total effects. The average difference between performance immediately after stimulation and right before simulation (immediate, d*n*post compared to d*n*pre, left bars), were greater in the anodal group (gray bars; *P* = 0.015). In contrast, effects between days (overnight, d*n*pre compared to d(*n*−1)post, central bars) were greater in the sham group (white bars; *P* = 0.034). Total enhancement of contrast perception (total, right bars) at day 5 (post), compared to baseline was superior on average (*P* = 0.047) in the anodal group than in sham. ^*^P < 0.05; error bars show SEM. *N* = 24 healthy subjects (12 sham group, 12 anodal group).

At the last day, the average total enhancement from baseline to day 5 post-stimulation was 0.9 dB in the anodal group (Figure 4); in other words, stimuli presented on day 5 could be detected at a 23% weaker luminance compared to stimuli presented at the beginning of the experiment (baseline). In the sham group, contrast sensitivity was enhanced by 0.2 dB (i.e., 5% weaker stimulus luminance).

We investigated whether these effects were based on enhancement immediately after stimulation (immediate effect, d*n*post−d*n*pre) or if there was an additional enhancement between days, such that subjects started at a higher contrast sensitivity level after a night of rest (overnight effect, d*n*pre−d(*n*−1)post; Figure 4).

The average difference between performance immediately after stimulation and right before simulation was greater in the anodal group (Figure 4; unpaired Student's *t*-test: *P* = 0.015). In fact, in the control group, the immediate effect of receiving the sham stimulation was a decrease in contrast sensitivity (see Figure 4). We interpret this as a fatigue effect, i.e., subjects became tired as a consequence of the procedure (Flammer and Niesel, 1984). If this is the case, tDCS not only counteracted the fatigue effect, but the stimulation duration and strength of tDCS was powerful enough to induce a positive immediate effect. The difference in total improvement (day 5 post-stimulation compared to baseline) between the anodal group and the sham group was significant (unpaired Student's *t*-test: *P* = 0.047, see Figure 4).

tDCS-induced enhancement of consolidation overnight has been observed previously (Brasil-Neto, 2012). Our analysis reveals a contrasting pattern between the two groups (Figure 4). Total enhancement of the anodal group was mainly based on immediate effects (unpaired Student's *t*-test: *P* = 0.015). Between days (overnight), performance of subjects in the anodal group regressed to a level below the post-stimulation level of the previous day. In contrast, overnight effects were significantly greater in the control group than in the anodal group (unpaired Student's *t*-test: *P* = 0.034): the sham group started at a slightly higher level pre-stimulation than post-stimulation the previous day. This suggests that subjects recovered overnight from the observed fatigue effect.

### Follow-up measurements after 2 and 4 weeks reveal long-lasting effects within the central visual field

**Figure 5:**
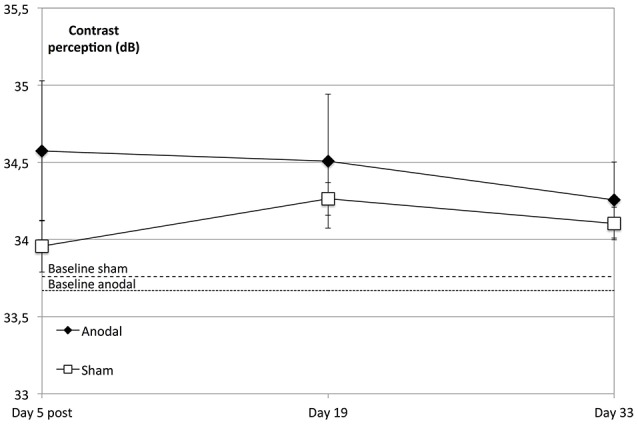
tDCS effects at follow-up dates. The enhancement in contrast perception at the follow-up measurements compared to baseline was not significant regarding the whole visual field (degrees 2–10) across all subjects (within-subject effect *time*: *F* = 1.798; *P* = 0.156) or between groups (between-subject effect *stimulation*: *F* = 1.073, *P* = 0.312). Over the study period and compared to the sham group (rectangle line), the anodal group (diamond line) showed no significant improvement of contrast sensitivity (interaction of within-subject effect *time* and between-subject effect *stimulation*: *F* = 2.390; *P* = 0.076). Compared to sham, tDCS-induced significant enhancement of contrast sensitivity (day 5 post) did not lead to a sharper decline on follow-up dates (*t* = 5, 19, 33; between-subject effect *stimulation*: *F* = 0.089, *P* = 0.768, within-subject effects *time*: *F* = 1.972, *P* = 0.151 and *interaction time* × *stimulation*: *F* = 0.244, *P* = 0.797). Baseline levels are the dotted (anodal) and dashed (sham) lines. Error bars show SEM. *N* = 24 healthy subjects (12 sham group, 12 anodal group).

We did not find a significant enhancement in contrast perception at the follow-up measurements compared to baseline regarding the whole visual field (degrees 2–10) across all subjects (*P* = 0.156) or between groups (*P* = 0.312). However, the anodal group showed an improved performance compared to that of the sham group over the whole visual field (degrees 2–10) and over the study's time span that was not significant (*P* = 0.076; Figure 5).

**Figure 6:**
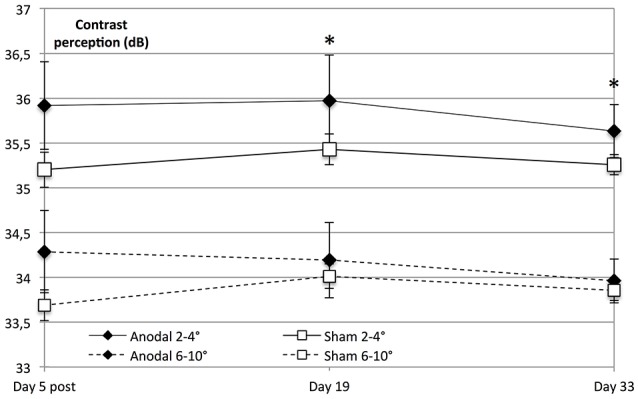
Significant lasting effect in the central visual field. Contrast sensitivity in the central visual field was significantly higher than peripheral sensitivity in both groups (within-subject effect *eccentricity*: *F* = 262.494, *P* < 0.001). Contrast perception on follow-up dates revealed that tDCS-induced enhancement of contrast perception was solely significant within the central visual field (anodal 2–4°; diamond line) on day 19 compared to baseline (degrees 2–4; *eccentricity* × *stimulation* × *time* day 19 to baseline: *F* = 7.338, *P* = 0.013) and day 33 compared to baseline (*F* = 6.144, *P* = 0.021), whereas there was no significant effect regarding the visual field as a whole (*stimulation* × *time* day 19 to baseline: *F* = 2.558, *P* = 0.124, and day 33 to baseline: *F* = 2.325, *P* = 0.140). ^*^P < 0.05; error bars show SEM. *N* = 24 healthy subjects (12 sham group, 12 anodal group).

Interestingly, on follow-up dates, the tDCS-induced enhancement of contrast perception was solely significant within the central visual field (degrees 2–4, day 19 to baseline, *P* = 0.013 and day 33 to baseline, *P* = 0.021), whereas there was no significant interaction regarding the peripheral visual field (day 19 to baseline, *F* = 2.555, *P* = 0.124 and day 33 to baseline, *F* = 2.341, *P* = 0.140; Figure 6).

Because of the retinotopic organization of V1, we considered it possible that tDCS had differential effects depending on eccentricity. We therefore investigated whether the enhancement of contrast sensitivity was spatially homogeneous within the examined visual field (10°). To this end, we compared central (2–4°) with peripheral (6–10°). We found that central visual contrast sensitivity was significantly higher than peripheral sensitivity in both groups (*P* < 0.001).

A repeated-measures ANOVA was performed to compare the development at the follow-up dates, revealing that the tDCS-boosted enhancement of contrast perception did not lead to significant differential decline of contrast perception after the stimulation period compared to that in the control group (day 5 post to days 19 and 33, *P* = 0.797; Figure 5).

## Discussion

This study is the first to provide evidence that anodal tDCS over the primary visual cortex induces long-lasting enhancement of contrast perception. We focused on the physiologically intact system of healthy subjects and showed that the degree of tDCS-induced improvement of contrast perception within 5 days was comparable in magnitude to the age-related decline of contrast perception over the course of 7 years (Hahn et al., 2009). Until now, it was unclear whether anodal tDCS could at all improve visual function.

Despite the popularity of tDCS in many areas of neuroscience, relatively few studies have investigated tDCS effects on the visual cortex (Antal et al., 2011; Olma et al., 2013) and, in contrast to stimulation of the motor cortex, tDCS application over the visual cortex has not been shown to induce comparable effects (Antal et al., 2001; Lang et al., 2007). Effects sustained beyond the end of the stimulation were limited to 15 min following 10 min of anodal tDCS of the visual areas, whereas 10 min of anodal tDCS over the motor cortex was able to induce sustained cortical excitability for up to 60 min (Nitsche and Paulus, 2001).

It is likely that the varying anatomical conditions are one reason for different tDCS effects. Because skull thickness determines the flow of current through the brain and thus the strength of tDCS effects (Datta et al., 2011; Giordano et al., 2017), one plausible reason for smaller tDCS effects over V1 compared to M1 can be seen in the relatively greater skull thickness and density of the occipital bone compared to the parietal bone (Voie et al., 2014; Zarghooni et al., 2016). The tDCS effects are also dependent on the distance and orientation of neuronal axons to the electrode (Paulus, 2003): the drift of membrane potential is higher and the tDCS effect more intense when current flow directs longitudinal to the neuronal axons, like in M1, than cross the axons (Nitsche et al., 2008). In contrast to M1, the V1 cells are mainly horizontally orientated and located deep in the occipital cortex (Dougherty et al., 2003). Thus, this aspect could be another reason for relatively weaker tDCS effects over V1.

In contrast to the discussed previous studies that showed only weak anodal tDCS effects on V1, our study substantiates the notion that tDCS over V1 induces long-lasting effects. We saw the first significant differences between anodal and sham group after the second stimulation (baseline to days 2–5 post-stimulation, see Figure 3). Thus, in contrast to M1, tDCS effects on V1 seem to be highly dependent on certain conditions, like a longer stimulation duration and repetitive tDCS application to overcome the anatomical barriers discussed above.

Contrast sensitivity significantly increased from baseline to day 5, demonstrating that subjects of the anodal group were able to detect darker luminance points with an average of 23% reduced stimulus luminance, compared to only 5% in the sham group. These findings indicate that tDCS was able to enhance the processing that is performed by a physiologically intact primary visual cortex.

To assess the size of the effect that we were able to induce via tDCS, let us compare our results with a perimetry study by Hahn et al. (2009). Hahn et al. investigated the extent to which there is an age-related decrease in contrast sensitivity in healthy subjects. Focusing on the central 8° of the visual field, they observed a linear age-related decline in contrast perception in healthy subjects starting at the age of 41 years. The improved contrast perception that we observed as a result of 5 days of anodal tDCS is comparable in magnitude to the age-related decline in contrast perception over the course of 7 years in healthy subjects. It is important to note that the decline in contrast sensitivity that Hahn et al. observed does not seem to be caused by age-dependent contrast processing in V1 but rather by age-induced impairment of the rod cells in the human retina. Still, our results suggest that it might be possible to at least partially offset this age-related loss of contrast sensitivity via noninvasive brain stimulation, since we only manipulated V1 activity and not rod cells.

We did not detect any tDCS-boosted consolidation overnight (i.e., in average d(*n* +1)pre ≤ d*n*post). This finding is in line with the results of a recently published study by Peters et al. (2013), which differ from the findings of other studies that investigated tDCS-induced overnight effects for different types of learning. One explanation might be that resting or sleep has been established to be beneficial particularly when human awareness is required and during declarative and procedural skill learning (Walker and Stickgold, 2004; Brown et al., 2009; Debarnot et al., 2009; Kandel, 2009; Doyon et al., 2011), while our relatively simple perimetry task predominantly requires implicit learning. Hence, an effect of sleep may be weak.

Importantly, our findings show that long-term tDCS effects were only significant within the central visual field—the retinal region with the highest contrast sensitivity (Skrandies, 1985). It is likely that tDCS-induced plasticity caused the improvement of vision and is responsible for the lasting enhancement of contrast perception within this visual field. Over the stimulation time (days 1–5), there was no differential enhancement of contrast perception between central and peripheral fields.

We argue that basic anatomical and structural conditions underly the long-term enhanced visual performance observed in this study. First, due to the topographic representation of the visual field in V1, the central visual field maps to the occipital pole and the adjacent brain area (i.e., areas close to the polarization electrode), while the parts of V1 that process the peripheral field run across the calcarine fissure in the depth of the brain (i.e., farther away from the polarization electrode; Horton and Hoyt, 1991). Consequently, compared to the brain regions that represent the peripheral field, a higher current density reaches the occipital brain region, possibly facilitating the synaptic strengthening within this region.

Second, relative to the peripheral visual field, a larger cortical area represents the central visual field in the cortex (Horton and Hoyt, 1991; Spillmann, 2014). The linear extent of the striate cortex to which each degree of the retinal visual field projects is called the magnification factor (Spillmann, 2014). In a positron emission tomography (PET) study (Fox et al., 1987), the magnification factor was investigated in the human striate cortex. Fox et al. (1987) observed that the neuronal response rate (i.e., the cerebral blood flow per mm) was higher for the central field, i.e., within the macular (0.1–1.5° with 3.4 mm/degree) and peri-macular region (up to 5.5° with 1.6 mm/degree) than for the peripheral field (5.5–15.5° with 0.9 mm/degree). Thus, in addition to the relatively higher current density reaching the areas that process the central 2–4 degrees of the visual field, these areas may be more susceptible to tDCS than are those representing the peripheral 6–10 degrees.

A potential explanation for the enhanced visual contrast perception that we observe is therefore that anodal tDCS triggers processes that alter the synaptic configuration within the visual cortex. This would be consistent with evidence that tDCS-induced long-term effects require LTP-dependent synaptic strengthening, as observed in studies using animal models (using direct current stimulation: Bindman et al., 1962; Creutzfeldt et al., 1962; Ranieri et al., 2012) as well in humans (for an overview, see Kim et al., 2010).

The underlying molecular mechanisms of tDCS-induced effects, especially following *repeated* tDCS application, appear similar to the innate learning processes of the visual cortex. These processes involve increased activation of NMDA receptors and higher concentration of BDNF (Fritsch et al., 2010; Kim et al., 2010), and they seem to be similar to the synaptic strengthening that gives rise to the consolidation of learned visual performance (for an overview, see Plow et al., 2012).

Interestingly, averaged across all subjects, there was a constant enhancement of contrast perception over the subsequent days (1–5), which might be the consequence of habituation to the test known to occur in repeated automated perimetry testing that follows the SITA standard (Yenice and Temel, 2005). Importantly, the enhancement of the control group over time was not significant. In contrast, subjects in the anodal group started, on average, on a significantly higher level of visual performance on following days (days 1–5), as evident from the significantly positive 24-h tDCS effects (Figure 2).

In this regard one could consider that tDCS enhances the experience-dependent plasticity that was observed in animal (DCS) studies (Cooke and Bliss, 2006; Cooke and Bear, 2010). These animal and also human (Frenkel et al., 2006) studies indicate that V1 plasticity is stimulus-specific. Like in these studies, we presented identical visual stimuli each day. Thus, it is possible that the daily presentation of the stimuli evoked a stimulus-specific response potentiation (SRP) in all subjects and that SRP was enhanced by daily anodal tDCS. To further address this hypothesis, tests with different stimuli would be necessary.

tDCS-induced performance enhancement from day 1 to day 5 did not lead to a steep reduction of contrast perception over the follow-up time points (days 19 and 33). However, testing performance of the anodal group peaked on the last day of the stimulation period (day 5 post). That is, while the anodal group showed superior performance on follow-up dates, contrast sensitivity decreased over those 2 weeks without tDCS application. In this regard, tDCS did not enhance consolidation processes once the stimulation block had ended.

Together, the findings of this study showed that tDCS improves visual perceptual performance directly after stimulation for about 24 h and up to a month later within the central visual field. Regarding these values as a first indicator for the time span over which an effect can be expected in the therapeutic application of tDCS to patients, our result suggests that it would be necessary to repeat tDCS application periodically.

As in a recently published study of Brückner and Kammer (2016), previous tDCS studies showed high inter-individual variability in the response to tDCS applied over the visual cortex. Given the high anatomic variability of the visual cortex in relation to the skull (Stensaas et al., 1974; Dougherty et al., 2003), the approach of referencing the stimulation electrode with the skull-based standard 10/20 EEG system (Brückner and Kammer, 2016) seems questionable but is widely used. In a recently published review about tDCS use within the sensory perceptual processing areas (Costa et al., 2015), only 6% of all 82 listed studies used an exact electrode position via MRI (3%) or transcranial magnetic stimulation (3%). However, it is likely that we were able to show long-lasting tDCS effects because we used MRI navigation to localize the individually optimal position directly over the calcarine sulcus.

## Conclusion

In sum, given the lack of previous studies investigating repeated tDCS application in the visual system of healthy controls, we provide novel insights by demonstrating long-lasting effects of tDCS over V1. In contrast to the majority of previous studies, we used a more precise method to determine the location of the polarization electrode on the surface using individual MRI data and navigation software. We attribute the observation of long-lasting effects to this method and the repetitive tDCS application. In contrast to short-lasting effects, long-lasting effects indicate plasticity. In this way, our results suggest that repetitive tDCS over V1 may be a promising neurorehabilitation tool for patients with chronic visual disability occurring after stroke. Importantly, the tDCS effects demonstrated by our study did not require an extensive, time-consuming, and effortful simultaneous visual training paradigm, such as VRT. This offers an important advantage in the development of rehabilitation programs for older patients who are more severely affected by diseases.

## Author contributions

JB, MO, AK, KI, and SB designed research. JB performed research. JB, MO, HG, AK, and SB analyzed the data. JB wrote the work. MO, HG, AK, KI, and SB critically revised the work, agreed to be accountable for all aspects of ensuring that questions related to the accuracy or integrity of any part of the work are appropriately investigated and resolved, and gave final approval of the version to be published

### Conflict of interest statement

The authors declare that the research was conducted in the absence of any commercial or financial relationships that could be construed as a potential conflict of interest.
